# Vitamin D Status Is an Independent Risk Factor for Global Cognitive Impairment in Peritoneal Dialysis Patients

**DOI:** 10.1371/journal.pone.0143782

**Published:** 2015-12-02

**Authors:** Gui-Ling Liu, Hai-Chen Pi, Li Hao, Dan-Dan Li, Yong-Gui Wu, Jie Dong

**Affiliations:** 1 Renal Division, the Second Hospital of Anhui Medical University, Hefei, Anhui, China; 2 Renal Division, Department of Medicine, Peking University First Hospital, Institute of Nephrology, Peking University, Key Laboratory of Renal Disease, Ministry of Health, Key Laboratory of Renal Disease, Ministry of Education; Beijing, China; Taipei Veterans General Hospital, TAIWAN

## Abstract

**Objective:**

Vitamin D (VD) deficiency is an independent risk factor for cognitive impairment (CI) in the general population, but VD status in peritoneal dialysis (PD) patients has not been investigated. In this study, we aimed to investigate the relationship between serum VD levels and global and specific cognitive functions in PD patients.

**Design and Setting:**

Cross-sectional study, simultaneously conducted at two PD centers.

**Patients:**

Clinically stable patients (n = 273) undergoing PD for at least 3 months were enrolled over a period of one year.

**Main outcome Measures:**

Demographic and comorbidity data were recorded, and routine biochemical parameters and serum 25-hydroxyvitamin D (25(OH) D) levels of overnight fasted patients were determined. Global cognitive function was assessed by the Modified Mini-Mental State Examination (3MS) score; executive function, by the trail making tests (Trails A and B); and immediate memory, delayed memory, and language ability by the Repeatable Battery for the Assessment of Neuropsychological Status (RBANS) sub-tests.

**Results:**

In the univariate analysis, serum 25(OH) D levels significantly correlated with 3MS scores (r = -0.139; P = 0.02), and Trail A (r = -0.188; P = 0.002) and B (r = -0.154; P = 0.01) completion times. In the multivariate analysis, 25(OH) D was found to be independently associated with global CI, but not with executive dysfunction. Serum 25(OH) D could not predict scores of immediate/delayed memory and language ability.

**Conclusions:**

VD deficiency is highly prevalent in PD patients and is an independent risk factor for global CI in this patient cohort.

## Introduction

Cognitive disorders have long been recognized as a complication of chronic kidney disease (CKD). The prevalence of cognitive impairment (CI) is as high as 27%–67% among patients with end-stage renal disease (ESRD) [1–4]. CI is an independent predictor of mortality [1] and overall survival [5] in dialysis patients. For peritoneal dialysis (PD) patients, home-care therapy and maintenance of normal cognitive function are especially important to be able to self-monitor and manage their treatment [6]. Therefore, it is critical to identify the risk factors for CI in this population cohort.

The mechanisms of CKD-dependent CI and dementia are multifactorial. Previous studies have identified several traditional and non-traditional factors associated with CI [7–10]. A few interventional studies on dialysis dose reported controversial findings [7, 11]. Elias et al. found that the estimated glomerular filtration rate (eGFR) is a strong predictor of global performance and CI in CKD patients free of dementia [7]. Kurella et al., on the other hand, suggested that albuminuria and eGFR together determine the incidence of CI [11]. These controversial findings indicate a need for identifying additional risk factors of CI as targets for potential intervention in dialysis patients.

Vitamin D deficiency has been identified as a risk factor of CI in the elderly population in the United States [12], Germany [13], and China [14]. One prospective study determined a longitudinal relationship between vitamin D status and cognitive decline in the elderly over a 6-year period [15]. This association between vitamin D status and CI prompted us to question the effect of vitamin D status on cognition in dialysis patients.

Only one study has to date identified an independent association between serum 25-hydroxyvitamin D [25(OH)D] levels and CI in hemodialysis patients [16]. The goals of our study were to determine the vitamin D status of PD patients with no history of stroke or neurodegenerative disease and identify its association, if any, with cognitive performance as determined by neuropsychological tests.

## Materials and Methods

### Participants

Two PD centers, namely, the Second Hospital of Anhui Medical University and the Peking University First Hospital, participated in this cross-sectional study. The two centers are equipped with professional doctors and nurses dedicated to providing dialysis care. All study investigators and staff members underwent a training program to learn the standard methods of assessment as needed for this study. A manual containing detailed instructions for data collection was distributed to all trainees. Data from each center were collected under a strict quality control framework and inspected and optimized to ensure the integrity and accuracy of the database maintained. The ethics committees of both hospitals approved the study. All patients provided written informed consent for their information to be collected.

Patients who underwent PD between May 2013 and May 2014 were enrolled in the study. The inclusion criteria were as follows: 1) age ≥18 years; 2) duration of PD ≥3 months; 3) clinical stability; and 4) ability to undergo all measurements and fill all questionnaires as required. Patients were excluded if they experienced acute cardiovascular events or trauma; had a systemic infection, active hepatitis, or cancer; or underwent surgery in the month before the commencement of the study. Patients with other conditions that could obstruct the progress of the study, such as severe visual impairment, language incompatibility, illiteracy, mental disturbance, and upper limb disability, were also excluded from the study. All the subjects received conventional glucose-based, lactate-buffered PD solution (Ultrabag; Baxter Healthcare, Guangzhou, China). A total of 315 patients receiving PD therapy at the two hospitals met the inclusion criteria. Seventeen patients refused to participate in the study, and 25 patients could not complete all assessments. Finally, 273 clinically stable patients with complete background data could be enrolled and their data analyzed.

### Study design

**Fig 1:**
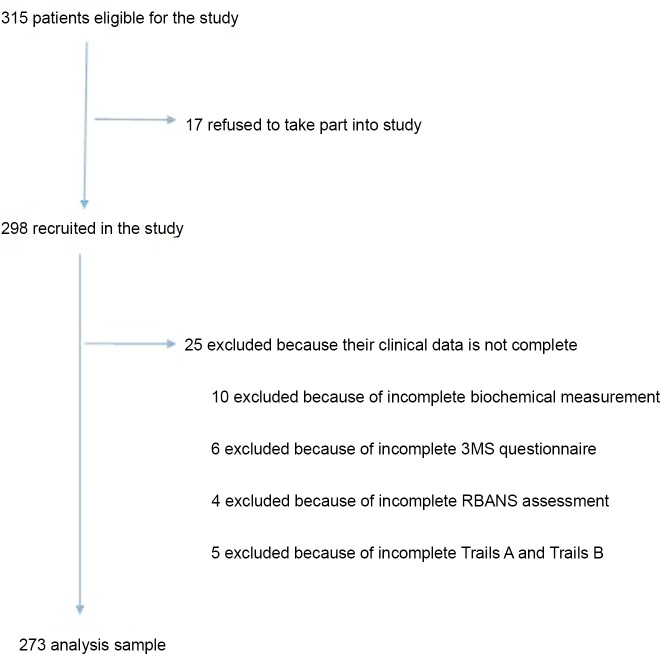
Flow of participant selection and exclusion process. A flow chart explaining the various steps from screening, to inclusion and exclusion, and final analysis, with the total number of patients lost during each step.

All patients in our study could visit a physician at least once every 3 months. After overnight fasting while continuing PD therapy, participants had their venous blood sampled for routine and biochemical measurements. Neuropsychological examination was conducted in a quiet environment by a specially trained research coordinator at a time when the participant believed they were at their best. To maintain quality and inter-rater reliability, testing was observed by the four research assistants after training. To limit subject fatigue, all testing was completed in less than an hour. A flow chart explaining the various steps from screening, to inclusion and exclusion, and final analysis, with the total number of patients lost during each step, as Fig 1.

### Clinical characteristics

Demographic characteristics, such as age, gender, education level, and factors contributing to comorbidities, such as dialysis duration, body mass index (BMI), presence of diabetes mellitus (DM) and cardiovascular disease (CVD), were recorded. The Eastern Cooperative Oncology Group (ECOG) scale scores were evaluated and patients were categorized as scores 0, 1, 2, 3, or 4 depending on their physical performance. The higher the score worse was the performance. The education level was recorded as the highest school grade at which a diploma was received, namely, elementary school or lower; middle school; high school; or above high school. CVD was said to be present if one of the following conditions was present: angina, class III–IV congestive heart failure as categorized according to the New York Heart Association classification, transient ischemic attack, history of myocardial infarction or cerebrovascular accident, and peripheral arterial disease [17].

### Biochemical parameters

Venous blood was drawn after overnight fasting from patients undergoing continuous PD to determine levels of routine and specific biochemical parameters. Intact parathyroid hormone (iPTH) and 25[OH]D levels were measured by chemiluminescence and chemiluminescence immunoassay, respectively. The instrument of 25[OH]D measured used was: Roche ECL e601. Precision: 2.7–6.8% (high-value, low). Detection range: 3.00–70.0ng / ml. Specificity: the cross-reactivity with serum 25-hydroxyvitamin D (25(OH) D) was 100%. And there was no cross-reaction with 1,25-dihydroxyvitamin D3, 1,25- hydroxy vitamin D2, vitamin D3, vitamin D2. Sensitivity: 4.01ng / ml (CV18.5%). Traceability: The detection method is traceable to LC-MS / MS method, and further traceable to NIST standards. Biochemical data including serum albumin (SA), triglyceride (TG), total cholesterol (TCHO), high-sensitivity C-reactive protein (HsCRP), and hemoglobin (Hb) were calculated as the mean of measurements taken over the three months preceding the study date. Biochemical profiles were investigated using an automatic Hitachi chemistry analyzer. HsCRP levels were determined by a nephelometric assay kit. Vitamin D deficiency was defined as a serum 25[OH]D level of < 10 ng/mL. Dialysis adequacy was defined as the total Kt/V calculated by collecting dialysate and urine over the course of 24 h to measure fluid and solute clearance.

### Neuropsychological status

Neuropsychological examination was conducted separately for each patient in a quiet environment by a trained research coordinator. To maintain quality and inter-rater reliability, test proceedings were observed by four trained research assistants. Thus, four medical personnel participated in the study as observers and completed a training program to ensure the integrity and accuracy of data assessment. To limit participant fatigue, all testing was completed in less than an hour.

The full battery of tests included the Modified Mini-Mental State Examination (3MS) to test global cognitive function and trail making tests A (Trail A) and B (Trail B) to test executive function, such as decision-making and processing speed [18]. The 3MS consists of a series of questions assessing a broad range of cognitive functions, including recall, execution, naming, orientation, writing, repeated imitation, and the ability to process speed. Global cognitive impairment was defined as a score of <80 on the 3MS test [19]. Executive dysfunction was defined as a Trail A score of >75 s or a Trail B score of >180 s [20]. In addition, the raw scores on subtests of the Repeatable Battery for the Assessment of Neuropsychological Status (RBANS) were used to assess immediate memory (list learning and story memory), delayed memory (list recall, list recognition, story recall, and figure recall), visuospatial skills (figure copy), and language ability (picture naming and semantic fluency). The raw scores were transferred to age-standardized T-score for all subtests of RBANS. The T scores less than 1 SD below the published mean in education-grouped Chinese population were identified as impaired for each test [21].

### Statistical analysis

Continuous variables were presented as means ± SD, except for duration of PD, iPTH, and HsCRP, which were presented as the median with interquartile range, owing to their highly skewed nature. Categorical variables were presented as proportions.

To determine predictors of cognitive function, univariate analysis was performed, and the potential risk factors of global and specific cognitive function were determined by the Spearman correlation analysis. Recognized risk factors, including serum 25(OH)D levels, obtained from the univariate regression analysis were used for multivariate regression analyses to determine the association of serum 25(OH)D levels and cognitive parameters of function independent of other confounders.

All probabilities were two-tailed, and the level of significance was set at 0.05. Statistical analysis was performed using the SPSS software for Windows, version 20.0 (SPSS Inc., Chicago, IL).

## Results

### Clinical characteristics and biochemical parameters

A total of 273 prevalent PD patients (136 men and 137 women; mean age, 53.58±14.06 years) were enrolled. Among them, 215 patients came from the Peking University First Hospital (with a mean age of 56.05±1.28 years, range 19~82 years), 63 were from the Second Hospital of Anhui Medical University (with a mean age of 44.47±1.47 years, range 18~77 years). Comparison of demographic data and clinical characteristics between patients from the two center reveal significant differences in age, gender (47.44% men VS 58.62%), the proportion of diabetes (33.48% VS 1.72%) and cardiovascular disease (28.37% VS 13.79%), hemoglobin ((114.04 VS 90.47)g/L), serum albumin ((39.02 VS 29.27) g/L),Triglycerides ((2.22 VS 1.34)mmol/L), corrected calcium ((2.39 VS 2.28)mmol/L) and intact parathyroid hormone (165.60 VS 272.00)pg/ml). The median serum 25(OH)D level was 9.85 ± 3.67 ng/mL(Table 1). According to the cut-off value of <10 ng/mL for deficiency, 59.91% (163/273) of patients were found to have vitamin D deficiency. In general, the study cohort had 49.81% men with a mean age of 53.58 ± 14.05 years and mean dialysis duration of 26.80 months (range, 10.85–55.38 months). The most frequent primary kidney disease was chronic glomerulonephritis (45.42%), followed by diabetic nephropathy (21.98%) and hypertensive nephropathy (10.26%). Most subjects (60.07%) were at least high school graduates. The mean BMI of 22.82 ± 3.46 kg/m^2^ was consistent with that of the general PD population in China [22]. The frequency of diabetes and CVD was 26.74% and 25.27%, respectively. The mean Hb, SA, and total Kt/V values were indicative of a relatively preserved nutritional status and dialysis adequacy (Table 1).

**Table 1:** Clinical characteristics of peritoneal dialysis patients.

	Values (n = 273)
Serum 25[OH]D (ng/ml)	9.85 ± 3.67
25[OH]D<10ng/ml(%)	59.91
Age (years)	53.58 ± 14.06
Male (%)	49.81
Dialysis duration (months)	26.80(10.85–55.38)
Primary kidney disease	
Diabetic nephropathy (%)	21.98
Hypertensive nephropathy (%)	10.26
Chronic glomerulonephritis (%)	45.42
Others (%)	17.95
Education level	
Elementary school or lower (%)	12.09
Middle school (%)	27.84
High school (%)	33.70
Above high school (%)	26.37
Normal physical performance (ECOG <2, %)	96.70
BMI (kg/m2)	22.81 ± 3.46
DM (%)	26.74
CVD (%)	25.27
Hb (g/L)	109.02 ± 15.94
Serum albumin(g/L)	36.94 ± 5.56
Triglycerides (mmol/L)	2.03 ± 1.32
Cholesterol (mmol/L)	4.63 ± 1.02
Corrected calcium	2.37±0.25
Phosphorus	1.64±0.41
iPTH (pg/mL)	179.80 (77.60–312)
HsCRP (mg/L)	3.22 (1.19–8.70)
Total Kt/V	1.86 (1.67–2.11)

ECOG: Eastern Cooperative Oncology Group; BMI: body mass index; DM: diabetes mellitus; CVD: cardiovascular disease; MAP: mean arterial pressure; Hb, hemoglobin; iPTH: intact parathyroid hormone, HsCRP: high-sensitivity C-reactive protein; Kt/V, urea clearance

## General and specific cognitive dysfunction

CI as determined by 3MS scores was observed in 22.34% patients (Table 2). The mean 3MS scores were 86.42 ± 10.95. The average Trail A and Trail B scores indicate that 49.08% participants had executive dysfunction. The scores on other subtests of RBANS, such as immediate and delayed memory, visuospatial skills, and language ability are shown in Table 2. We divided the study population into 2 groups, 25-OH-D <10 ng/ml and ≥ 10 ng/ml groups and analyzed the demographic characteristics, biochemical parameters and cognitive status. We found there were significant statistical differences in the Trail A and visuospatial skill scores between two groups.

**Table 2:** Parameters of cognitive function of peritoneal dialysis patients.

	Total (n = 273)	25-OH-D<10 ng/ml (n = 163)	25-OH-D≥10 ng/ml (n = 110)
3MS (score)	86.42 ± 10.95	85.53±1.24	87.75±8.24
CI (%)	22.34	23.93	20.00
Trail A (s)	65.00(50.00–90.50)	65.00(52.00–105.00)*	60.00(45.00–86.25)
Trail B (s)	156.00(108.50–236.50)	166.00(110.00–240.00)	149.00(105.00–212.75)
Executive dysfunction (%)	49.08	49.07	49.09
Immediate memory (score)	34.08 ± 9.72	34.03±1.02	34.15±9.00
Delayed memory (score)	43.92 ± 9.51	43.26±1.04	44.91±7.91
Visuospatial skill (score)	16.32 ± 3.96	15.91±4.44*	16.92±3.04
Language ability (score)	28.82 ± 5.87	28.56±6.11	29.21±5.51

3MS: modified Mini-Mental State Examination; CI: cognitive impairment; Trail A: Trail making test A; Trail B: Trail making test B

*P < 0.05, compared with the 25-OH-D≥ 10 ng/ml group

### Association between serum 25[OH]D level, clinical characteristics, and cognitive function

Univariate analysis (Table 3) revealed that serum 25[OH]D levels significantly correlated with 3MS scores and completion times of Trails A and B, but not with any of the RBANS scores. We analyzed the correlation between 25-OH-D and subscales of 3MS. We still did not find any association between vitamin D status and parameters of specific cognitive functions such as executive function, memory and language. Moreover, age, education level, presence of DM, and SA levels were significantly associated with general cognitive function and almost all specific cognitive parameters. Furthermore, ECOG scores and TCHO levels were associated with completion times of Trails A and B; Hb levels, center and season, with 3MS scores; and Hs-CRP levels, with completion time of Trail A. Univariate analysis of the association between serum 25[OH]D and cognitive function of subscales of 3MS.revealed that serum 25[OH]D levels significantly correlated with 3MS' visuospatial skills and language ability, but not with any other aspects of 3MS (Date not shown).

**Table 3:** Univariate analysis showing correlation between demographic data, clinical and laboratory parameters, and cognitive function.

	3MS	Trail A	Trail B	Immediate memory	Delayed memory	Visuospatial skill	Language ability
	r	r	r	r	r	r	r
25[OH]D	0.139*	-0.188*	-0.154*	0.060	0.096	0.109	0.084
center	-0.143*	0.080	-0.041	0.003	-0.086	-0.041	-0.099
age	-0.345**	0.407**	0.516**	-0.396**	-0.416**	-0.323	-0.237**
male	-0.04	0.121*	0.139*	0.056	-0.019	0.080	-0.043
Dialysis duration	-0.009	0.052	0.074	0.032	0.038	0.008	0.049
Education level	0.399**	-0.329**	-0.230**	0.229**	0.304**	0.279**	0.290**
ECOG score	-0.140	0.087**	0.220**	-0.221**	-0.232**	-0.120*	-0.106
BMI	-0.150*	0.097	0.123*	-0.116	-0.142*	-0.124*	-0.019
DM	-0.245**	0.283**	0.285**	-0.230**	-0.222**	-0.265**	-0.131*
CVD	-0.088	0.113	0.094	-0.160**	-0.144*	-0.166**	-0.146*
MAP	0.072	-0.118	-0.209**	0.091	-0.128	0.131	0.072
HB	0.143*	-0.073	0.040	0.044	0.097	0.080	0.161**
Serum Albumin	0.229**	-0.196**	-0.138*	0.116	0.168*	0.133*	0.140*
Triglyceride	-0.023	-0.022	0.066	-0.004	0.000	0.006	-0.018
Cholesterol	-0.064	0.148*	0.179**	0.015	-0.041	-0.055	0.008
Corrected calcium	0.018	0.040	0.063	0.026	-0.004	-0.114	0.028
Phosphorus	0.023	-0.089	-0.035	-0.010	0.030	0.111	0.085
iPTH	0.032	-0.006	-0.022	0.051	0.017	0.146*	0.045
HsCRP	-0.115	0.167**	0.148*	-0.130*	-0.113	0.146*	-0.110
Total Kt/V	-0.139*	-0.045	0.006	0.150*	0.142*	0.153*	0.072
Season	-0.149*	0.073	-0.045	-0.008	-0.103	-0.037	-0.104

ECOG: Eastern Cooperative Oncology Group; BMI: body mass index; DM: diabetes mellitus; CVD: Cardiovascular disease; MAP: mean arterial pressure; Hb, hemoglobin; iPTH: intact parathyroid hormone, HsCRP: high-sensitivity C-reactive protein; Kt/V, urea clearance

*P < 0.05

**P < 0.01

Table 4 shows the results of multivariate linear regression analysis performed with serum 25(OH)D level and other potential confounders to identify risk factors of poor 3MS scores and delayed completion times for Trails A and B. After adjusting for age, education level, BMI, DM, SA level, Hb level, total Kt/V, center and season, serum 25(OH)D was found to be independently associated with general cognitive function as evaluated by 3MS scores (β = 0.187; 95% CI = 0.067–0.307; P = 0.002). The association between serum 25(OH)D and completion time of Trail A (β = -0.176; 95% CI = -0.806–0.453; P = 0.582) and that of Trail B (β = - 0.176; 95% CI = -1.980–1.628; P = 0.847) disappeared after multivariate regression analysis. Regression analysis after including the dialysis centers variable shows that the relationship between serum 25 (OH)D and 3MS score still exists. Moreover, linear regression analysis revealed a non-linear correlation between vitamin D status and immediate memory, delayed memory, visuospatial skills, and language ability. Moreover, serum 25(OH)D level could not predict the risk of CI or executive dysfunction whether it as a continuous variable or as a categorical variable (Table 5).

**Table 4:** Multivariate linear regression analysis the association between serum 25[OH]D and cognitive function of subscales of 3MS.

	3MS	Trail A	Trail B	Immediate memory	Delayed memory	Visuospatial skill	Language ability
	β	β	β	β	β	β	β
25[OH]D	0.187**	-0.176	-0.176	—-	—-	—-	—-
center	-3.770	—-	—-	—-	—-	—-	—-
age	-0.288**	1.015**	3.259**	-0.256**	-0.229**	-0.053**	-0.100**
male	—-	9.213	18.110	—-	—-	—-	—-
Education level	5.515**	-24.688**	—-	4.139**	4.043**	1.738**	2.995**
ECOG score	—-	4.203	5.312	-0.645	0.648	0.006	—-
BMI	-0.280	—-	4.584*	—-	-0.286	-0.089	—-
DM	-2.973*	15.408*	21.437	-2.226	-1.598	-1.691**	—-
CVD	—-	—-	—-	0.509	0.123	-0.429	-1.176
MAP	—-	—-	0.165	—-	—-	—-	—-
HB	0.034	—-	—-	—-	—-	—-	0.045
Serum Albumin	0.060	-1.589**	-1.059	—-	0.364**	0.135**	-0.031
Cholesterol	—-	3.246	-2.186	—-	—-	—-	—-
HsCRP	—-	0.212	0.317	-0.052	—-	-0.024	—-
Total Kt/V	1.150	—-	—-	3.304*	1.154	0.079	—-
Season	-2.869	—-	—-	—-	—-	—-	—-

ECOG: Eastern Cooperative Oncology Group; BMI: body mass index; DM: diabetes mellitus; CVD: Cardiovascular disease; MAP: mean arterial pressure; Hb: hemoglobin; HsCRP: high-sensitivity C-reactive protein; Kt/V: urea clearance

*P < 0.05

**P < 0.01

**Table 5:** Logistic regression analysis of vitamin D as a predictor of CI and executive dysfunction using different groups of vitamin D levels.

	CI ^a^		Executive dysfunction ^b^	
	OR	P	OR	P
25[OH]D as continuous variable	0.976	0.201	0.979	0.195
25[OH]D as categorical variable				
25[OH]D ≤10ng/ml	ref	—-	ref	—-
25[OH]D >10ng/ml	1.155	0.677	0.419	0.053

CI: cognitive impairment

a: adjusted for age, Education level, body mass index; diabetes mellitus; hemoglobin; serum albumin and urea clearance.

b: adjusted for age, gender, Education level, Eastern Cooperative Oncology Group; body mass index; diabetes mellitus; mean arterial pressure; serum albumin; cholesterol and high-sensitivity C-reactive protein.

## Discussion

In our PD patient cohort with no history of stroke or neurodegenerative diseases, a high prevalence of CI and executive dysfunction was observed. These prevalence rates for PD patients are higher than prevalence rates previously reported for hemodialysis patients [23]. Parameters of specific cognitive decline, such as executive dysfunction, immediate and delayed memory, visuospatial skills, and language ability, were also substantially impaired in our PD patients. These results are consistent with those of previous studies involving CKD, hemodialysis, and PD patients [3, 24, 25].

Consistent with a previous study, vitamin D deficiency was found to be prevalent in a significant proportion of PD patients [26, 27]. The possible reasons of high prevalence of vitamin D dificiency include chronic renal failure, dietary restrictions, loss of vitamin D in the peritoneal effluent and decreased exposure to sunlight [27]. A significant relationship between serum 25(OH) levels and general cognitive function was further verified after adjusting for demographic characteristics, physical capacity, and clinical parameters. However, Vitamin D status could not independently predict the risk for impairment in executive function as evaluated by the completion time of Trail A. Moreover, no association was found between vitamin D status and impairment of memory function, visuospatial skills, or language ability.

The mechanism by which vitamin D alters cognitive function is hitherto unclear. Vitamin D was found to improve cognitive function in rats with hepatosteatosis via its anti-inflammatory and antioxidant properties [28]. It also elicits certain neuroprotective effects by reducing serum levels of anti-neurofilament 160 and anti-myelin basic protein, thereby preventing neuronal degeneration and death and axonal degeneration [29]. Moreover, vitamin D was found to increase cerebrospinal fluid levels of amyloid β and brain tissue volumes [30]. Cognitive function usually declines with ageing in the general population. Consistent with this observation, several studies have identified a direct correlation between vitamin D deficiency and CI in the elderly population in various countries [12–15].

We found a significant, but weak, correlation between vitamin D status and global CI (r = 0.139), which is relatively low compared to that reported by Przybelski and Binkley [31]. Moreover, Peterson et al. reported a correlation coefficient of 0.24 between vitamin D status and general cognitive function in independently living older adults aged >70 years [32]. Several factors could have contributed to the weak correlation in PD patients in our study. First, in CKD, several parameters, such as dialysis duration [33], renal function [7, 11], anemia [10], and vascular factors [9] contribute to general CI. Thus, we believe that the predictive power of vitamin D status for CI in PD patients is low. Interestingly, we found relatively stronger correlation between cognitive function and albumin levels (r = 0.229), education level (0.399), and age (r = -0.345). Secondly, vitamin D levels was markedly low (59.71% participants exhibiting serum 25(OH)D levels of <10 ng/mL) in our population as compared to that reported(1.8%–7.3%) in previous literatures [12, 34, 35]. This may explain why we did not observed a significant relationship between vitamin D deficiency and cognitive impairment in those studies [12, 34, 35].

Furthermore, we did not find any association between vitamin D status and parameters of specific cognitive functions, such as memory and language. This finding is consistent with that of Minasyan et al [36], but contradictory to that of other researchers [25, 28]. But in our study, we found that serum 25 (OH) is relevant to global cognitive function. We added the corrected calcium, phosphate and parathyroid hormone (PTH) in the correlation analysis and did not find any relationship between them and vitamin D. These findings indicate that the influence of serum 25-OH-D on cognition function is independent on calcium and PTH in PD population. As we all know most peritoneal dialysis patients were treated at home, their physical activities are much less than general population. Less sun exposure might be associated with their vitamin D deficiency. At the same time, PD patients at home have less contact with other people, which is not good for cognitive function. From these perspectives, the relationship between vitamin D and cognitive dysfunction could be influenced by less physical activity and less contact with other people. Although we evaluated physical activity by Eastern Cooperative Oncology Group (ECOG) in this study and did not find its confounding effect on that relationship, we are aware that more studies including physical activity, social and family support should be designed to determine whether vitamin D independently correlate to cognitive dysfunction. Thus, the detailed mechanisms by which vitamin D elicits its effects on cognition warrant further investigation.

Our study findings have several important clinical implications. Because vitamin D status can act as a sensitive indicator of cognitive function, serum vitamin D assessment could be included in the routine evaluation of patients on dialysis therapy. Moreover, it would be interesting to investigate whether vitamin D supplementation can improve cognitive function in PD patients. In the general population, some evidence exists for the beneficial effects of vitamin D intake on cognition. A randomized, double-blind, placebo-controlled trial involving women aged 35–60 years and men aged 45–60 years found that midlife vitamin D intake exhibited longitudinal and domain-specific effects on cognition in the context of aging [37]. Furthermore, a pilot study conducted in 43 white older outpatients identified that weekly dietary intake of vitamin D for six months improved cognitive performance [38]. With regard to CKD patients undergoing hemodialysis, a variable regime of cholecalciferol has been verified to be effective and safe, but no target for improvement in cognitive function has been set [39–41]. Additional studies will be required to elucidate the association between vitamin D deficiency and CI in the dialysis population and to identify the efficacious vitamin D dose that can achieve improvement in cognitive function.

Our study had several advantages. It was the first study to examine the association between vitamin D status and cognitive function in PD patients. Our cognitive battery included a wide range of tests that encompass a broad spectrum of cognitive domains such as execution, memory, and language function. Our study also considered some recognized confounders of CI in the general population and patients on dialysis, such as MAP, BMI, TCHO, HsCRP, total Kt/V, and physical activity. In addition, our cohort could be considered relatively generalizable because the demographics of patients refusing consent was relatively similar to that of patients who provide consent.

Our study did have some limitations. First, because of its cross-sectional nature, we could not determine whether vitamin D deficiency is a pathogenic factor or a risk biomarker of CI. Second, serum vitamin D levels were measured only at baseline and not monitored over an extended duration. Future studies should be designed to evaluate cognitive function and serum 25(OH)D levels longitudinally in PD patients. Moreover, a randomized control study to explore whether cognitive function would be improved by correction of this metabolic disorder would help determine the therapeutic potential of vitamin D specifically in dialysis patients. Third, our study did not investigate the mechanism of vitamin D action in dialysis patients. Potential confounding effects of different pathogenic factors affecting the association between vitamin D status and CI could not be identified. Fourth, the finding that low vitamin D level is a predictor of global CI was not substantiated with evidence from imaging studies. Future studies should focus on validating the association between vitamin D status and CI by eliminating potentially confounding factors and substantiating clinical evidence with imaging study results.

In conclusion, this is the first study to investigate the relationship between vitamin D status and cognitive function in PD patients. The prevalence of CI and vitamin D deficiency was found to be very high in this population. Moreover, vitamin D status was found to be independently associated with general cognitive function in this population. These data support the hypothesis that vitamin D deficiency plays one of causes of CI in PD patients. Further interventional studies will be required to determine whether correcting vitamin D deficiency improves CI and overall survival of PD patients.
